# Gene Organization, Expression, and Localization of Ribotoxin-Like Protein Ageritin in Fruiting Body and Mycelium of *Agrocybe aegerita*

**DOI:** 10.3390/ijms21197158

**Published:** 2020-09-28

**Authors:** Ilaria Baglivo, Sara Ragucci, Paolo D’Incecco, Nicola Landi, Rosita Russo, Franco Faoro, Paolo V. Pedone, Antimo Di Maro

**Affiliations:** 1 Department of Environmental, Biological and Pharmaceutical Sciences and Technologies (DiSTABiF), University of Campania ‘Luigi Vanvitelli’, 81100 Caserta, Italy; ilaria.baglivo@unicampania.it (I.B.); sara.ragucci@unicampania.it (S.R.); nicola.landi@unicampania.it (N.L.); rosita.russo@unicampania.it (R.R.); PaoloVincenzo.PEDONE@unicampania.it (P.V.P.); 2 Department of Food, Environmental and Nutritional Sciences (DeFENS), University of Milan, 20133 Milan, Italy; paolo.dincecco@unimi.it; 3 Department of Agricultural and Environmental Sciences—Production, Landscape, Agroenergy (DiSAA), University of Milan, 20133 Milan, Italy; franco.faoro@unimi.it

**Keywords:** *Agrocybe aegerita*, cDNA extraction, immunolocalization, signal peptide, ribotoxin-like proteins, protein routing

## Abstract

The edible mushroom *Agrocybe aegerita* produces a ribotoxin-like protein known as Ageritin. In this work, the gene encoding Ageritin was characterized by sequence analysis. It contains several typical features of fungal genes such as three short introns (60, 55 and 69 bp) located at the 5′ region of the coding sequence and typical splice junctions. This sequence codes for a precursor of 156 amino acids (~17-kDa) containing an additional *N*-terminal peptide of 21 amino acid residues, absent in the purified toxin (135 amino acid residues; ~15-kDa). The presence of 17-kDa and 15-kDa forms was investigated by Western blot in specific parts of fruiting body and in mycelia of *A. aegerita*. Data show that the 15-kDa Ageritin is the only form retrieved in the fruiting body and the principal form in mycelium. The immunolocalization by confocal laser scanning microscopy and transmission electron microscopy proves that Ageritin has vacuolar localization in hyphae. Coupling these data with a bioinformatics approach, we suggest that the *N*-terminal peptide of Ageritin (not found in the purified toxin) is a new signal peptide in fungi involved in intracellular routing from endoplasmic reticulum to vacuole, necessary for self-defense of *A. aegerita* ribosomes from Ageritin toxicity.

## 1. Introduction

Ageritin is a monomeric protein isolated from *Agrocybe aegerita* fruiting bodies that inhibits protein synthesis in vitro [1]. This enzyme catalyzes the cleavage of a specific phosphodiester bond of larger rRNA. The specific hydrolysis due to its ribonuclease action occurs in the Sarcin Ricin Loop (SRL), which is necessary for the binding of EF-G or EF-2 elongation factors on the ribosome in prokaryotes and eukaryotes, respectively [2]. This rRNA endonuclease enzymatic activity releases a 460 nt-fragment (α-fragment) at 3′ end of the 28S RNA [3]. The diagnostic α-fragment is also released from a family of specific ribonucleases known as ribotoxins isolated from ascomycetes fungi, whose prototype is α-sarcin [4,5]. In this framework, Ageritin is the first example of ribotoxin isolated from basidiomycetes fungi, although sequence similarity among Ageritin, α-sarcin, and other members of ascomycetes ribotoxin family is low [6]. In light of this, Ageritin represents the first member of novel specific ribonuclease family retrieved in basidiomycetes for which our group proposed the name of ribotoxin-like proteins [1,6,7].

Ageritin consists of 135 amino acid residues (~15 kDa) with a single free cysteinyl residue [8], differently from ascomycetes ribotoxins, which contain two intramolecular disulphide bonds [6]. The pI of this toxin is basic (≥9.5), and it is not glycosylated [1]. Structurally, by homology modeling, Ageritin exhibits α/β fold with a structural core consisting of an antiparallel β-sheet with an adjacent long α-helix, similar to both ascomycetes ribotoxins and fungal RNases. The β-sheet in Ageritin structure has an orthogonal orientation with respect to the α-helix, unlike the other classic ribotoxins [6,9]. The catalytic residues located in a pocket of the β-sheet are Asp68, Asp70, and His77 forming the catalytic triad [6,9]. Ageritin was found to inhibit protein synthesis in a rabbit reticulocyte lysate with an IC**_50_** value of 133 pM [1] and is able to release the α-fragment when exerts its action on ribosomes isolated from several sources [i.e., rabbit, yeast, bacteria (*E. coli* and *Micrococcus lysodeikticus*), or mold (*Trichoderma asperellum* and *Penicillum digitatum*) [1,10,11]. In addition, this enzyme possesses endonuclease activity on the viral RNA from Tobacco Mosaic Virus (TMV) and plasmid and genomic DNAs, which, differently from ascomycetes ribotoxins, depends on the presence of metal ions such as Zn^2+^ and Mg^2+^ [9,10]. Indeed, Ageritin is a metal-dependent ribotoxin-like protein as confirmed by the presence of binding sites for Ca^2+^, Mg^2+^ and Zn^2+^ through isothermal titration calorimetry binding studies [9].

The physiological role of this enzyme is not yet found, while several studies show that Ageritin exerts: (i) antiproliferative activity toward CNS model cell lines (SK-N-BE(2)-C, U-251, C6, and SH-SY5Y cells), COLO 320, HeLa, and Raji cell lines by promoting apoptosis [1,10]; (ii) antifungal activity against *T. asperellum* [12] and *P. digitatum* molds [10]; and (iii) strong toxicity against *Aedes aegypti* larvae, yet not against nematodes [13].

Due to the inhibition of translation and apoptotic activities of Ageritin, this toxin is a novel possible biological tool useful both as therapeutic agent in biomedicine and as biopesticide in a sustainable agriculture as reported for ascomycetes ribotoxins [14,15]. In addition to that, an appropriate research on this specific ribonuclease could better clarify the strategies developed by fungi against antagonists and helps to elucidate the large possessed armamentarium implicated in fungal defense [16].

Taking into account the above, the aim of the present study was to characterize the structure of the *ageritin* gene including exons/introns patterns by sequence analysis. Moreover, considering that mature *ageritin* gene presents an additional coding nucleotide sequence at the 5′ that translates for a peptide of 21 amino acid residues at the *N*-terminal region compared to purified Ageritin [1,6], the presence of this toxin was investigated in specific parts of fruiting body and in mycelium of *A. aegerita* by Western blot analyses. Furthermore, the subcellular localization of Ageritin was studied by microscopy immunolocalization techniques to verify extracellular secretion or compartmentalization (e.g., vacuoles) of this toxin in *A. aegerita* mushroom to avoid the potential toxicity against itself. Finally, bioinformatics analyses were carried out to find out a possible functional role of the additional *N*-terminal peptide in Ageritin structure.

## 2. Results

### 2.1. Analysis of ageritin Gene

In the *Agrocybe aegerita* AAE-3 gene database collecting all the identified coding sequences derived from the analysis of the *A. aegerita* AAE-3 genomic sequence [17], the sequence encoding for Ageritin protein is identified with the gene ID: AAE3_01767 [13], Figure S1. The genomic sequence containing the coding sequence ID AAE3_01767 is located in the scaffold 12 in a region spanning from the nucleotide in position 672,601 to that in position 673,490.

The alignment between the genomic sequence 672,601–673,490 and the coding sequence AAE3_01767 reveals that *ageritin* gene is composed of three exons and two introns (Figure S1). The three exons are 236, 39, or 255 nucleotides long, while the two introns are 55 and 69 nucleotides.

**Figure 1:**
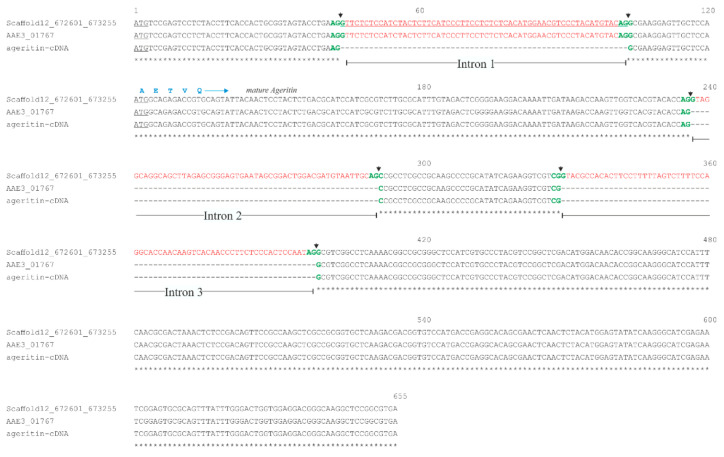
Sequence alignment of the *ageritin*-cDNA, AAE3_01767 and scaffold 12 genomic region 672601-673490. The ATG codon is underlined in black; the exons are reported in black, introns in red (new intron red and underlined, see main text), the splice junctions in green. The arrows indicate the splicing site. In blue the first amino acid residues of mature Ageritin. Fully conserved bases are indicated by (*).

On the basis of the *ageritin* gene sequence, we designed a couple of primers to amplify by Polymerase Chain Reaction (PCR)the *ageritin* gene using genomic DNA from *A. aegerita* as a template. As shown in Figure S2, the sequence of the PCR product has been aligned with the genomic region in the scaffold 12 (nucleotides 672,601–673,490) showing that no nucleotide differences are present between the PCR product that we obtained and the *ageritin* gene in *A. aegerita* AAE-3 genomic sequence [17]. Then, we used the same couple of primers to amplify the *ageritin* cDNA in order to get its sequence. The sequence of the *ageritin* cDNA obtained was aligned with that of *ageritin* coding sequence AAE3_01767. The alignment reveals the presence of an additional intron of 60 bp, which was not originally identified in the *ageritin* coding sequence AAE3_01767 (Figure 1). This new intron splits the first exon of the AAE3_01767 into two sequences (Figure 1) which constituted two exons, the first one of 43 nucleotides and the second one of 133 nucleotides. Therefore, *ageritin* gene presents three introns shorter than 80 bp located at the 5′ region of the coding sequence as already reported for fungi [18].

Finally, the three introns present canonical 5′-GU-…-AG-3′donor-acceptor splice sites previously described as intron consensus elements [19], which have been highlighted in Figure 1.

### 2.2. Expression of Ageritin in Fruiting Bodies and Mycelium

The characterization of *ageritin* gene reveals the presence of a region encoding for an additional peptide of 21 amino acid residues located at *N*-terminal region of Ageritin protein (Figure S3; hereafter _+21_Δ-Ageritin, ~17-kDa), which was not found by our group during Ageritin purification (~15-kDa) from *A. aegerita* fruiting bodies. Tayyrov and co-workers have reported in a recent work the cloning and expression of a cDNA coding for _+21_Δ-Ageritin, derived from total RNA of fruiting primed mycelium [13]. Moreover, these authors concluded that the additional 21 amino acid residues at *N*-terminal region of Ageritin could have a regulatory function or the lack of it in purified protein could be due to proteolysis events during the purification procedure.

**Figure 2:**
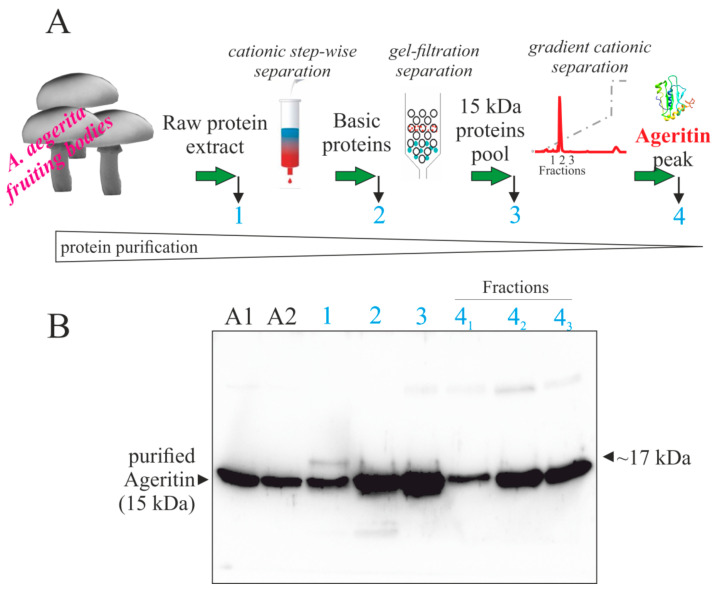
Western-blot analysis of soluble proteins from *A. aegerita* fruiting bodies during purification procedure (scheme **A**). In (**B**): lane 1, 10 μg of total protein extracted from total fruiting bodies; lanes 2, 3, and 4 (1, 2, 3 fractions) 2.5, 1.0, and 0.5 μg of total soluble proteins after cationic step-wise chromatography, gel-filtration separation, and cationic chromatography eluted with a linear gradient, respectively. The samples were separated by SDS-PAGE with β-mercaptoethanol and subjected to Western blot analysis using the rabbit antiserum against Ageritin. SDS-PAGE was carried out in 15% polyacrylamide separating gel. A1 and A2, 100 and 50 ng of purified Ageritin as control [1], respectively.

In this framework, we decided to verify the expression of Ageritin in both fruiting bodies and mycelium from *A. aegerita* by Western blot analysis using anti-Ageritin rabbit polyclonal antibodies. First, we carried out the purification steps necessary to purify Ageritin from fruiting bodies [1], and we checked the purified protein by Western blot analysis after each step of purification (Figure 2).

**Figure 3:**
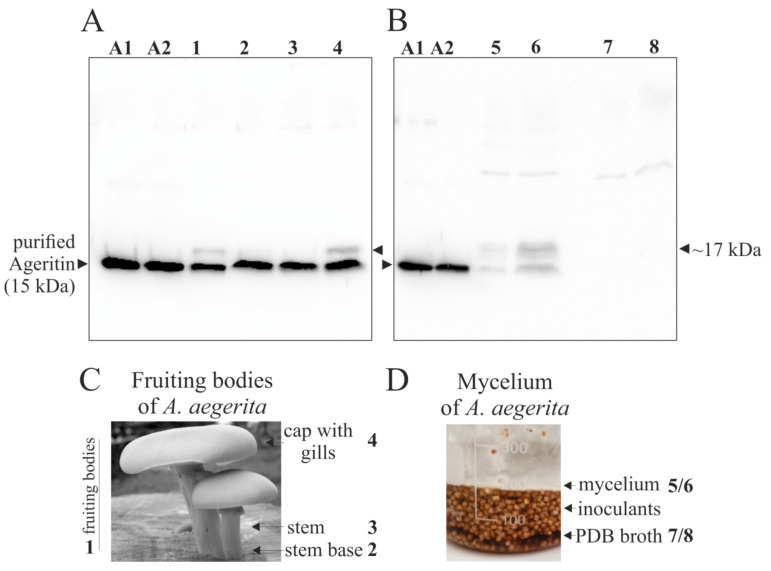
Western blot analysis of proteins extracts from *A. aegerita* fruiting bodies or mycelim. (**A**) 10 μg of total proteins extracted from total fruiting bodies were loaded into lane 1, from base steams into lanes 2 and 3 and from cap with gills into lane 4. In (**B**), 10 or 50 μg of total proteins extracted from mycelium were loaded into lanes 5 and 6, respectively. The same amount of total proteins present in potato dextrose broth (PDB) medium used for mycelia growth were loaded into lanes 7 and 8, respectively. As a control, in both panel A and C, lanes A1 and A2 were loaded with 100 and 50 ng of purified Ageritin, respectively [1]. The samples were separated by SDS-PAGE with β-mercaptoethanol and analyzed by Western blot using the rabbit antiserum antibody against Ageritin. SDS-PAGE was carried out in 15% polyacrylamide separating gel. In (**C**,**D**), schematic representation of single parts of *A. aegerita* mushroom and mycelium cultured in PDB medium are shown.

This analysis displays that from raw protein homogenate to the last step of Ageritin purification by ion-exchange chromatography, a single anti-Ageritin-reacting band (~15-kDa) is present, although a slight cross-reactive band with an electrophoretic migration of ~17-kDa is observable in raw protein homogenate (lane 1, Figure 2). Thus, it is evident that if the 17-kDa band represents _+21_Δ-Ageritin, it is not the most abundant cross-reactive component in *A. aegerita* fruiting bodies homogenate and does not decrease during purification procedure. Furthermore, an additional check has been performed by subjecting to Western blot the raw protein extracts obtained homogenizing total fruiting bodies (lane 1, Figure 3A) or single parts (stem base, stem, and cap with gills, lane 2, 3, and 4, respectively; Figure 3A).

As expected, a main anti-Ageritin-reacting band with an electrophoretic migration of ~15-kDa is detected in both fruiting bodies and single fungi parts as well as when using the purified Ageritin (lanes A1 and A2; Figure 3A), whereas a slight anti-Ageritin reacting band with an electrophoretic migration of ~17-kDa is present only in homogenate protein extracts from both fruiting bodies and cap with gills. Finally, a similar approach by Western blot was carried out to verify the presence of Ageritin in mycelia (lanes 5 and 6; 10 and 50 μg of total protein extracted, respectively; Figure 3B) and in concentrated potato dextrose broth (PDB) medium used for mycelia growth (lanes 7 and 8; 10 and 50 μg of soluble proteins, respectively; Figure 3B). In this case, two anti-Ageritin-reacting bands with an electrophoretic migration of 15 and 17-kDa appeared with a similar amount (~50%) in mycelia extract, while there are not reacting bands in PDB medium, indicating that Ageritin is not exported out of mycelium cells.

In light of these data, we can reasonably say that the mature protein product of *ageritin* gene in *A. aegerita* fruiting bodies is the 135 amino acid residues form of Ageritin lacking 21 additional amino acid residues at the *N*-terminal region. This additional peptide could be a regulatory region given the toxicity of the ribotoxin against its own ribosomes or is implicated in unconventional transport/secretion processes [20,21].

**Figure 4:**
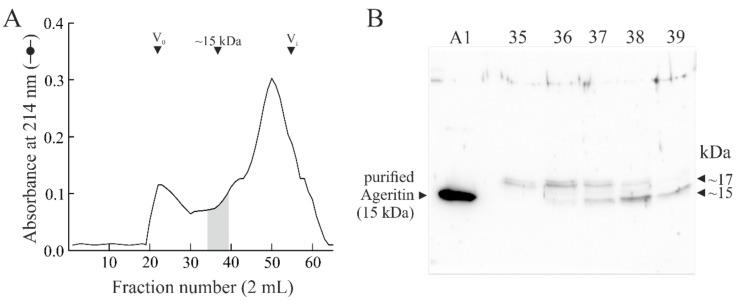
(**A**) Elution profile from the gel-filtration chromatography on a HiLoad 16/60 Superdex 75 column obtained for mycelium basic proteins. V_0_ and V_i_, void and included volumes, respectively. (**B**) Western blot analysis of fractions with an elution volume about 15-kDa (highlighted in grey). 0.5 μg of proteins eluted in fraction 35, 36, 37, 38, and 39 was loaded in respective lanes. Fractions aliquots were separated by SDS-PAGE with β-mercaptoethanol and subjected to Western blot analysis using rabbit antiserum against Ageritin. SDS-PAGE was carried out in 15% polyacrylamide separating gel. A1 and A2, 100 and 50 ng of Ageritin as control [1], respectively.

Considering the presence of a 17-kDa band with an electrophoretic migration similar to the theoretical weight of the _+21_Δ-Ageritin (16,910.17 Da) detected through Western blot analysis in mycelium, we decided to perform an analytical purification from *A. aegerita* mycelium following the purification protocol, previously reported [11] and schematically represented in Figure 2A. In Figure 4A, the elution profile of analytical gel filtration is reported (for more details see Figure S4), while in Figure 4B the Western blot analysis of the fractions with an elution volume of ~15-kDa is shown.

**Figure 5:**
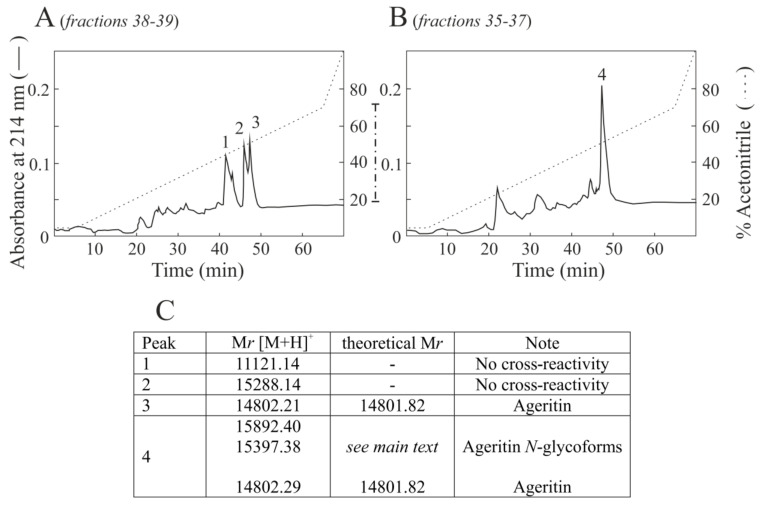
RP-HPLC separation of 38–39 (**A**) and 35–37 (**B**) cross reactive fractions obtained after gel-filtration separation of basic proteins extracted from *A. aegerita* mycelium. In (**C**), relative molecular mass (M*r*) of peaks containing cross-reactive electrophoretic bands using the rabbit antiserum against Ageritin. The average molecular masses values obtained by MALDI-TOF MS are reported as [M + H^+^]^+^.

The Western blot analysis displays that two anti-Ageritin-reacting bands were present with an electrophoretic migration of 17-kDa (fraction 35, 36 and 37) and 15-kDa (fraction 38 and 39). Thus, fractions containing 15-kDa (38 and 39) and 17-kDa (35, 36 and 37) proteins bands were pooled, and considering the low proteins amount, subjected to RP-HPLC to obtain desalted samples and their enrichment (Figure 5). The cross-reactive protein peak (peak 3, Figure 5A) after RP-HPLC of 15-kDa pool analyzed by Matrix-Assisted Laser Desorption/Ionization-Time Of Flight (MALDI-TOF) mass spectrometry (MS) displays a relative molecular mass (M*r*) of 14802. 21, which is in good agreement with the M*r* of Ageritin amino acid sequence (14801.82; ([M + H^+^]^+^) [6]. On the other hand, the single cross-reactive protein peak (peak 4) after RP-HPLC of 17-kDa (Figure 5B) contains different relative molecular masses (i.e., 15,892.40 and 15,397.38), which do not correspond to theoretical relative molecular mass of _+21_Δ-Ageritin (16,911.17 Da; [M + H^+^]^+^). In this framework, the results shows that the apparent 17-kDa cross-reactive protein band in mycelium is not Ageritin with the additional peptide of 21 amino acid residues at *N*-terminal region (Figure S2). Moreover, our opinion is that the band with an electrophoretic migration of 17-kDa is due to Ageritin micro-heterogeneity given its cross-reactivity with anti-Ageritin antibodies. One possibility could be the presence of posttranslational modifications such as *N*-linked glycosylation considering that Ageritin sequence contains two potential *N*-glycosylation consensus motifs at N79 and N118 (Figure S2) [22,23].

### 2.3. Localization of Ageritin in A. aegerita Fruiting Bodies

**Figure 6:**
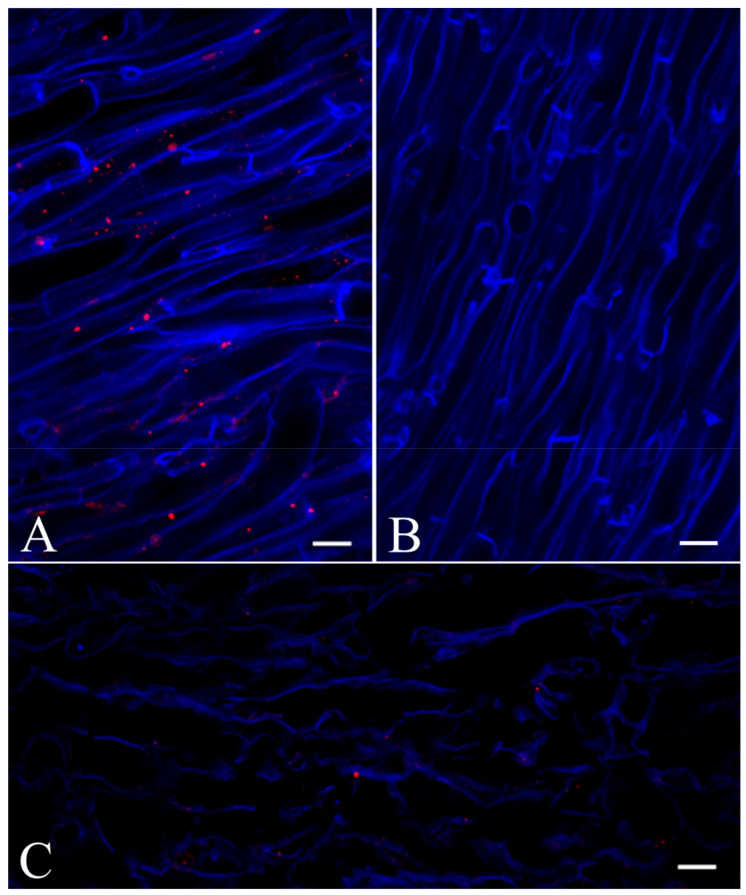
Immunolocalization of Ageritin on cryo-sections of *A. aegerita* (**A**,**B**) and of *Agaricus campestris* (**C**) by confocal laser scanning microscope. A clear positive signal is present in form of dots inside hyphae of *A. aegerita* (**A**) but not in *A. campestris* (**C**) that does not synthesize this compound, except for a few background; labelling is also lacking in (**B**), a negative control in which the rabbit antiserum against Ageritin is missing. All bars are equals to 20 µm.

The immunolocalization of Ageritin in *A. aegerita* fruiting body was first studied by Confocal Laser Scanning Microscopy (CLSM) with the same polyclonal antibodies used for Western blot analysis and a secondary antibody labelled with Cy-3 fluorochrome. Labelling was detected as roundish fluorescence spots of different sizes, with a maximum diameter of 1–2 µm inside hyphae, usually leaning cell wall, but apparently not over cell wall or in the periplasm (Figure 6A). No signal was found when the primary antibody was omitted (Figure 6B) or when labelling was performed on sections of *Agaricus campestris* L., a related species that does not produce Ageritin, indicating the labelling specificity of the probe (Figure 6C).

**Figure 7:**
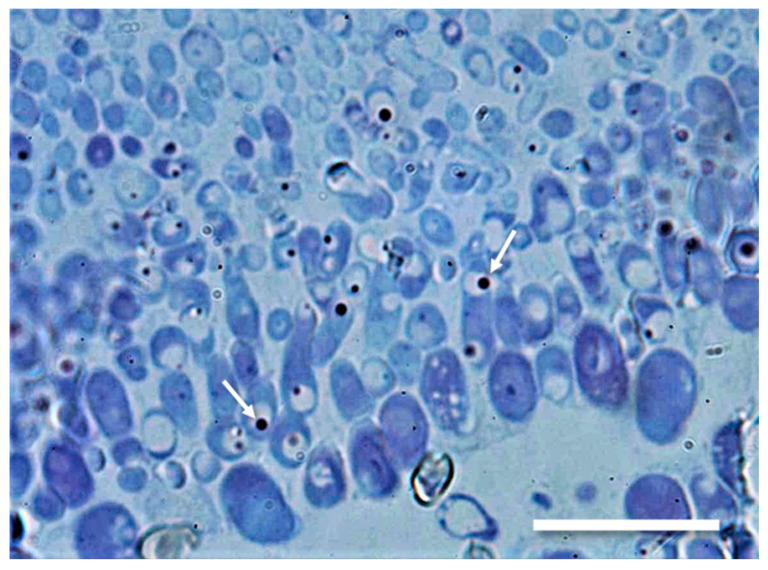
Semithin section stained with toluidine blue of *A. aegerita* terminal hyphae in a fruiting body observed by bright field microscopy: numerous dark dots (arrows) are present inside the vacuoles. Part of this section has been processed for immunolabelling and examined by TEM (see Figure 8). Bar is 10 µm.

For a more precise localization of Ageritin inside hyphae, a Transmission Electron Microscopy (TEM) analysis was performed focusing on hyphae close to basidia, richer in cytoplasm and organelles. A first survey on semithin sections of this tissue examined by light microscope, after staining with toluidine blue, showed the presence of dark dots (Figure 7), clearly visible inside hyphae vacuoles reminiscent of typical plant protein bodies [24].

**Figure 8:**
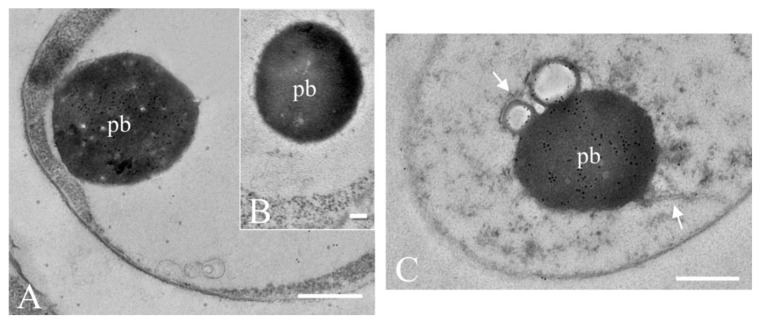
Immunogold labelling of ultrathin sections of *A. aegerita* with the rabbit antiserum against Ageritin showing the presence of numerous 10 nm gold particles within an electron dense protein-like body (pb) inside the vacuole of an hyphae (**A**). No gold particles are present in labelling control with a pre-immune serum (**B**). The protein body (pb) seems to be originated inside the ER (**C**, arrows). Bars are 500 nm in (**A**,**C**) and 200 nm in (**B**).

Ultrathin sections of these hyphae examined by TEM revealed the same roundish bodies observed by CLSM and light microscopy, mostly present in the cell vacuole and closely resembling typical protein bodies (Figure 8A). These various bodies were not homogenously electron dense and only some areas were intensely labelled by the immunogold probe, suggesting they were formed by other proteins/compounds besides Ageritin (Figure 8A). No labelling was present in control sections (Figure 8B). In some instances, protein bodies seemed to be originated inside the endoplasmic reticulum as ER membranes were seen around them (Figure 8C). However, it was not possible to find out direct evidence of their route from the ER to vacuole, where they appear to be compartmentalized. Except for a few background, no labelling was also detected either on hyphae wall or in the periplasm, confirming the intracellular localization of Ageritin, which is possibly sequestered into the vacuole to avoid its toxic activity on ribosomes.

### 2.4. N-Terminal Signal Peptide of Ageritin

Many proteins present a signal peptide that directs them outside of the cell or to certain intracellular compartments after protein synthesis. For this reason, the cell synthesizes preforms, which are then matured through proteolytic processes [25]. In addition, for some enzymes, it has been reported that the presence of an additional *N*-terminal peptide absent in mature form has the ability to modulate the enzyme function by inhibiting its activity until the proteolytic removal of the peptide [26,27].

**Figure 9:**
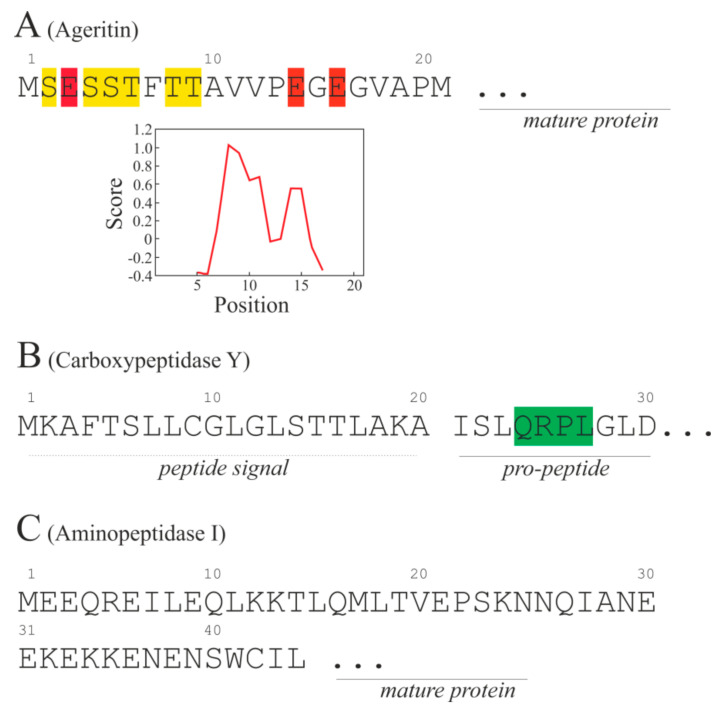
*N*-terminal amino acid signal peptide of Ageritin (**A**) and both *N*-terminal sequence of Carboxypeptidase Y (**B**; AC: P00729) and Aminopeptidase I (**C**; AC: P14904) from yeast implicated in vacuolar intracellular pathway. In A, the Kyte–Doolittle–Hydropathy plot of Ageritin *N*-terminal signal peptide is also reported. In yellow are highlighted tyrosinyl and serinyl residues, in red glutamyl residues. In green consensus amino acid sequence.

Therefore, considering the intracellular localization of Ageritin in vacuole by immunolocalization technique, the *N*-terminal peptide _−21_ MSESSTFTTAVVPEGEGVAPM _−1_ (2127.37 Da; pI = 3.67) might play a role as a signal peptide for vacuolar destination (Figure 9A), although no known consensus sequences for subcellular localization were found by bioinformatics approach. From a structural point of view, this peptide contains a greater amount of both serinyl and threonyl residues (3 for each; 28.5% on total), followed by glutamyl residues (3; 14.3%) and no basic amino acids. On the other hand, the Kyte–Doolittle–Hydropathy plot (Figure 9A) shows the presence of a hydrophobic region (position 6–15) at the center of the sequence, which could play a key role in the intracellular signaling vacuolar pathway for Ageritin in this edible mushroom.

In fungi, the vacuole function is analogous to the mammalian lysosome for which it is an acid compartment rich in hydrolytic enzymes [28]. In addition, the vacuoles are involved in many cellular processes such as metabolites transport and storage, cytosol acidification, and ion homeostasis. Most of the hydrolases retrieved in vacuoles follow an intracellular pathway that requires the synthesis of proenzymes and subsequently the routing through the endoplasmic reticulum (ER) and Golgi complex for vacuolar delivery, while other enzymes follow an independent pathway consisting in a direct way from cytoplasm to vacuole. Among the examples of this process, carboxypeptidase Y [29] and aminopeptidase I [30] are the most studied showing different prototypes of *N*-terminal signals that allow vacuolar protein delivery in yeast. In particular, carboxypeptidase Y presents an *N*-terminal signal peptide followed by a consensus sequence present in the pro-peptide consisting of four contiguous amino acid residues (QRPL; Figure 9B) necessary for ER and Golgi pathway [29]. On the other hand, aminopeptidase I contains an amino-terminal 45-amino acid pro-peptide (Figure 9C) lacking of a consensus signal sequence but with an amphipathic α-helix followed by a β-turn and another α-helix, forming a helix-turn-helix structure used for an independent pathway from cytoplasm to vacuole [30].

In this framework, the *N*-terminal signal peptide of Ageritin (Figure 9A) is different from both the reported examples; therefore, it is possible that this peptide is a novel example of a signal peptide for vacuolar delivery in edible mushrooms, as retrieved for Ageritin through TEM experiments (see above).

## 3. Discussion

In this work, we adopted a plethora of biochemical approach on both fruiting bodies and mycelium to obtain information about gene and expression of Ageritin, the first ribotoxin-like protein from the edible mushroom *A. aegerita*.

A comparison between the *ageritin* coding sequence AAE3_01767 and the cDNA encoding for Ageritin protein found in *A. aegerita* reveals that *ageritin* gene contains three introns instead of two as originally predicted by genome sequence analysis. Our finding is in agreement with the recent results of Tayyrov et al. [13] reporting the presence of this new intron at the 5′ region *ageritin* gene coding sequence. The analysis of the *ageritin* gene shows that short introns interrupt the coding sequence at the *N*-terminal amino acid region of the protein and present typical splice junctions as reported for other fungi. Moreover, the nucleotide coding sequence confirms the presence of a region encoding for a putative additional peptide (21 amino acid residues) at the *N*-terminal of this toxin not retrieved in purified Ageritin. Considering a detectable electrophoretic migration difference between Ageritin with (named _+21_Δ-Ageritin; ~17-kDa) and without (~15-kDa) this addition of *N*-terminal peptide, we have decided to verify the presence of the two forms in fruiting bodies and mycelium and during protein purification (necessary to exclude a possible non-specific proteolytic cleavage) by Western blot analysis. Overall, our data show that 15-kDa form is the only form present in raw protein extract and during the purification steps in both fruiting bodies and mycelium. Moreover, the principal mushrooms structure that synthesizes Ageritin is the fruiting body (2.5 mg/100 g; [11]), since Ageritin is retrieved in low amount (~40 ng/100 g) in mycelium.

Subsequently, considering the additional *N*-terminal peptide of Ageritin as a potential signal for specific compartmentalization or for extracellular localization in hyphae cells, since the Ageritin toxin could damage the ribosomes of its producing cells, a classical approach of immunolocalization by CLSM and TEM in addition to bright field microscopy was carried out to clarify the localization of Ageritin in *A. aegerita* hyphae. In vivo, all the techniques show that Ageritin is an intracellular protein clearly present in the hyphae cells. Moreover, detailed observation through TEM analysis revealed that Ageritin has a vacuolar localization (stroma) where it is segregated in highly electron-dense structures, already visible in the endoplasmic reticulum, very similar to plant protein bodies [31]. In this framework, the data demonstrate an intracellular pathway of Ageritin that foresees endoplasmic reticulum, probably Golgi apparatus and, finally, the vacuole destination [32]. Moreover, not having found the 17-kDa form in hyphae, the *N*-terminal additional peptide probably is quickly cleaved from the protein core by proteolytic process during translation before route starting. This peptide has physicochemical properties different from the others widely characterized in basidiomycetes known to date. Therefore, in light of this, the additional peptide at the *N*-terminal region of Ageritin might be a new specific fungal signal for vacuolar localization.

Further studies will be performed to verify the Ageritin toxicity in human diet since the *A. aegerita* mushroom is widely consumed.

## 4. Material and Methods

### 4.1. Materials

The sources of the chemicals used in this work have been previously indicated [1,9], and most of them were obtained from Sigma-Aldrich (St. Louis, MO, USA).

### 4.2. Mushroom Samples

The fruiting bodies and mycelia used in this work for protein purification, total RNA extraction, and subcellular localization were obtained using the commercial inoculant provided from ‘ITALSPAWN ITALIA S.r.l.’ di Valentino e Massimo Sartor, Onigo di Pederobba (Treviso, Italy). The society identifies the product as *Agrocybe aegerita* YY (AA YY). Homogeneous preparation of Ageritin used as reference protein was purified either from *A. aegerita* fruiting bodies collected on poplar stumps in rural Aversa sites (Aversa, Caserta, Campania Region in Southern Italy) or from fruiting bodies grown by the ‘FUNGHI GROTTE DI COSTOZZA’ s.s.a. di A. Rigoni & G. Dal Pozzo (Vicenza, Veneto region Italy; http://www.funghigrottedicostozza.com). The ‘FUNGHI GROTTE DI COSTOZZA’ company uses only the inoculants supplied by ‘ITALSPAWN ITALIA S.r.l.’ (code AA YY) and kindly provided us both the fruiting bodies and the inoculants used in this work.

Edible parts of fruiting bodies were washed and stored at −80 °C, until further analyses, while the inoculants to obtain mycelia were grown at 26 °C in PDB medium. In particular, mycelia of *A. aegerita* were prepared by growing the commercial inoculant (~10 g) in 125 mL of PDB medium in autoclaved cylindrical containers (pyrex graduated glass beakers, capacity 500 mL) at 26 °C for 8 days without shaking. The resulting mycelia were centrifuged at 3000× *g* to remove the PDB medium and washed with 5 mM Na-phosphate, pH 7.2 containing 0.14 M NaCl until use [12].

### 4.3. Protein Purification

Homogeneous preparation of Ageritin was achieved as previously described [1,11]. The homogeneity of protein was verified by using SDS-PAGE and RP-HPLC. The same procedure was performed for analytical purification of basic protein from mycelium. Only the gel-filtration separation was carried out with different conditions (see size-exclusion chromatography).

### 4.4. Analytic Size-Exclusion Chromatography

Gel filtration was performed on an AKTA Purifier 10 System (Ge Healthcare Srl, Milano, Italy) using a HiLoad 16/60 Superdex^TM^ 75 prep grade column (volume, 120 mL; Ge Healthcare). The column was equilibrated and eluted with 5 mM Na/P containing 0.3 M NaCl, pH 7.2 (flow rate 1 mL/min) by monitoring the absorbance at 214 nm. The elution volumes of the following standard proteins were used for column calibration: BSA (M, 66 kDa) horse heart myoglobin (M, 17 kDa), ubiquitin (U, 8.5 kDa).

### 4.5. RP-HPLC and MALDI-TOF Analysis

Prior to MALDI-TOF MS analysis both 15-kDa and 17-kDa proteins obtained from mycelium purification were desalted by RP-HPLC [33]. In particular, the separation was obtained on a Breeze Instrument (Waters, Sesto San Giovanni, Milano, Italy) using a C-4 column (0.46 × 25 cm, 5 micron; Phenomenex, Castel Maggiore, Bologna, Italy), at a flow-rate of 0.5 mL min^−1^. Protein elution were obtained using linear gradients of 0.1% TFA (solvent A) and acetonitrile containing 0.1% TFA (solvent B) from 5 to 65% of solvent B over 60 min, monitoring the absorbance at 214 nm [34]. The relative molecular masses (M*r*) of protein were determined by using a MALDI-TOF micro MX spectrometer (Waters, Manchester, UK) in linear mode, as previously reported [35].

### 4.6. Western Blot Analysis

*A. aegerita* fruiting bodies (3.5 g), single parts (cap with gills, stem and ring; 3.5 g), or the mycelium (1.5 g) were homogenized by T 10 basic ULTRA-TURRAX (IKA^®^- Werke GmbH & Co. Staufen, Germany); for 5 min at 4 °C in 5 mM Na-phosphate, pH 7.2 containing 0.14 M NaCl, while PDB medium was concentrated in an Amicon cell concentrator (MWCO 3 kDa; Sigma-Aldrich) for further analyses. After clarification (60 min at 15,000× *g* at 4 °C), the protein content of samples was determined by the method of Bradford using Bio-Rad protein assay (Bio-Rad, Milan, Italy). Total protein extracts (10 μg) were separated by SDS-PAGE on a Mini-Protean II mini-gel apparatus (Bio-Rad), using 6% (*w/v*) stacking polyacrylamide gel and 15% (*w/v*) separation gel. Separated proteins were transferred onto nitrocellulose membrane (filter type 0.45 μm HATF; Millipore, Burlington, Massachusetts, USA) by electroblotting with Mini Trans-Blot Cell (Bio-Rad). Finally, the blot was probed with the anti-Ageritin polyclonal rabbit antibody (Bio-Fab research, Rome, Italy) as a primary antibody (dilution 1:1000). Following incubation with the anti-rabbit HRP-conjugated antibody (Bio-Rad, 1:3000), protein bands were revealed by adding the Clarity^TM^ Western ECL substrate (Bio- Rad) and acquired by using the ChemiDoc XRS System (Bio-Rad).

### 4.7. On-Line Bioinformatics Tool

For subcellular localization prediction, a search was performed with WoLF PSORT program available on-line (https://wolfpsort.hgc.jp/). The Kyte–Doolittle–Hydropathy plot was obtained using the ExPASy Protscale tool (https://web.expasy.org/protscale/). To obtain the computation of various physical and chemical parameters for proteins [(molecular weight, theoretical pI, amino acid composition, extinction coefficient, estimated half-life, instability index, aliphatic index and grand average of hydropathicity (GRAVY)] ProtParam tool was used (https://web.expasy.org/protparam/).

### 4.8. RNA Retrotranscription and Sequencing of PCR Product

Total RNA was extracted from *A. aegerita* mycelium (code AA YY) using Quick-RNA Fungal/Bacterial Miniprep Kit (Zymo Research). The RNA concentration was calculated by spectrophotometric absorbance measured at 260 nm. Ratio A**_260_**/A**_280_** and A**_260_**/A**_230_** were calculated for checking RNA purity; RNA integrity was checked by electrophoresis on 1.5% agarose gel. An amount of 1.0 μg of the total RNA extracted from mycelium was retrotranscribed by using SensiFAST cDNA Synthesis Kit (Bioline). An amount of 5 μL of cDNA were used to amplify *ageritin* cDNA by PCR using the Bestaq™ DNA Polymerase (abm) and 25 pmol of primers designed on the basis of the *ageritin* coding sequence AAE3_01767 [6] (forward primer: 5′-CAGCATATGTCCGAGTCCTCTACCTTCACC-3′; reverse primer: 5′-CGGAATTCTCACGCCGGAGCCTTGCCCG-3′); RT- sample was prepared as a control.

The thermal profile of PCR for ageritin cDNA amplification consisted of initial denaturation for 4 min at 95 °C followed by 94 °C for 30 s, 55 °C for 30 s, and 72 °C for 1 min (40 cycles), with a final extension at 72 °C for 7 min. PCR product from cDNA template was gel-purified using QIAquick Gel Extraction Kit (QIAGEN, Milano, Italy), the concentration was calculated by spectrophotometric absorbance measured at 260 nm, and a sample was sequenced by Sanger method.

The *ageritin* cDNA from *A. aegerita* mycelium (code AA YY) was gel-purified using QIAquick Gel Extraction Kit (QIAGEN), the concentration was calculated by spectrophotometric absorbance measured at 260 nm, and a sample was sequenced by Sanger method.

The same couple of primers mentioned above were used to amplify the *ageritin* gene by PCR (same thermal profile as described before). The template for this PCR was genomic DNA from *A. aegerita* (code YY) used for all the experiments in this study. The gel-purified PCR product was sequenced by Sanger method.

### 4.9. Preparation of Samples for Confocal Laser Scanning Microscopy (CLSM)

Mushroom tissues were cut to 10 µm sections using a Leica CM 1850 cryostat [Leica Instruments, Buccinasco, Italy]. The sections were laid on polylysine-covered slides, fixed in cold acetone for 5 min, and dried [36]. They were then immersed in a 1% dilution of bovine serum albumin (BSA) in 10 mM saline phosphate buffer pH 7.2 (PBS) to prevent non-specific binding. The procedure was repeated three times, and slides were washed with PBS after each treatment with BSA. They were then incubated for 1 h with primary anti Ageritin antibodies diluted 1:1000 in PBS containing 1% BSA. Unbound antibodies were washed-out with PBS, and the slides incubated for 1 h in the dark with Cy3-labelled secondary goat anti-rabbit antibodies (Jackson ImmunoResearch Lab; Cambridge House, UK) goat anti-rabbit IgG (H + L) and diluted 1:50 in PBS containing 1% BSA.

Negative controls consisted of: 1. mushroom tissue from *A. aegerita* fruiting bodies incubated with secondary antibodies only; 2. sections of a taxonomically related mushroom *Agaricus campestris* L. lacking Ageritin and incubated with both primary and secondary antibodies. Sections were inspected with an inverted CLSM (Nikon A1+, Minato, Japan) equipped with a HeNe laser. Autofluorescence of samples were obtained by exciting at 409 nm and the emission filter was set at 430–490 nm, while Cy3-labelled secondary antibody was excited at 560 nm and the emission filter was set at 565–615 nm.

### 4.10. Light and Transmission Electron Microscopy (TEM) and Immunogold Labelling

Small pieces of mushroom tissues (~2 mm × 2 mm) were fixed in a fixative solution (glutaraldehyde 1%, paraformaldehyde 4% in 0.1 M phosphate buffer; Agar Scientific, Stansted, UK) for 2 h at 4 °C [37]. Samples were washed with the same buffer for 1 h. Dehydration was carried out in a series of ethanol solutions prior to embedding in London Resin White (EMS, Hatfield, PA, USA) followed by curing at 60 °C for 24 h. Semithin sections (1–2 µm thick) were cut from blocks, stained with 2% toluidine blue in water and examined by an Olympus BX50 light microscope (Olympus, Tokyo, Japan). Ultrathin sections, 70 nm thick, were cut from the same blocks and incubated at room temperature for 15 min in an incubation buffer containing 50 mM PBS, 1% BSA, and 0.01% Tween 20 (both Aurion, Wageningen, The Netherlands) and, afterwards, in the same primary anti-Ageritin serum used for Western blot (Bio-Fab research, Rome, Italy) diluted 1:500 in the incubation buffer for 2 h at 38 °C. The sections were thoroughly washed in the same buffer for 20 min and, afterwards, incubated at 38° C for 1 h in 10-nm colloidal-gold labelled secondary antirabbit serum (diluted 1:20 in the incubation buffer) that had been raised in goat (Thermo Fisher Scientific, Rodano (MI), Italy).

Negative controls consisted of: 1. sections incubated with a pre-immune primary antibody followed by the secondary gold-conjugate antibody; 2. sections incubated with the secondary antibody only. After washing in 50 mM PBS and distilled water, sections were stained with uranyl acetate and lead citrate and examined with a TEM Leo 912ab (Zeiss, Oberkochen, Germany).

## Supplementary Materials

Supplementary Materials can be found at https://www.mdpi.com/1422-0067/21/19/7158/s1.
