# Supplementary material for: Gene Organization, Expression, and Localization of Ribotoxin-Like Protein Ageritin in Fruiting Body and Mycelium of Agrocybe aegerita
Source: Int J Mol Sci. 2020 Sep 28;21(19):7158. doi: 10.3390/ijms21197158 (PMC7582721; doi:10.3390/ijms21197158)
Supplement: Supplementary file 1 [file ijms-21-07158-s001.pdf]

```
                           1                                                                                               100
Scaffold12_672601_673490   AGGCGAGAAAAGAAACATAAGACCGCACTCAATGTGAAGGTGATGAATGGTGACTGTCAAAGAAACTTGGGTCATAGGATGAACAAAAGTTTATACCAAC

                         101                                                                                               200
Scaffold12_672601_673490   TTTCGAATTTATGGAAGACCTCTGGGGGAGCCTCCGTGAAGTGCAAACGCACATAAAGAGAAGAGGGAGGTCCTTGAACACGAAAGCCCAGCCCAGAGCT

                         201                                                                                               300
Scaffold12_672601_673490   CTCCCAACCACAGGCTCACAAAGCTGTCCTACACAATGTCCGAGTCCTCTACCTTCACCACTGCGGTAGTACCTGAAGGTTCTCTCCATCTACTCTTCAT
AAE3_01767                 -----------------------------------ATGTCCGAGTCCTCTACCTTCACCACTGCGGTAGTACCTGAAGGTTCTCTCCATCTACTCTTCAT
                                                              *****************************************************************

                         301                                                                                               400
Scaffold12_672601_673490   CCCTTCCTCTCTCACATGGAACGTCCCTACATGTACAGGCGAAGGAGTTGCTCCAATGGCAGAGACCGTGCAGTATTACAACTCCTACTCTGACGCATCC
AAE3_01767                 CCCTTCCTCTCTCACATGGAACGTCCCTACATGTACAGGCGAAGGAGTTGCTCCAATGGCAGAGACCGTGCAGTATTACAACTCCTACTCTGACGCATCC
                           ****************************************************************************************************

                         401                                                                                               500
Scaffold12_672601_67349    ATCGCGTCTTGCGCATTTGTAGACTCGGGGAAGGACAAAATTGATAAGACCAAGTTGGTCACGTACACCAGGTAGGCAGGCAGCTTAGAGCGGGAGTGAA
AAE3_01767                 ATCGCGTCTTGCGCATTTGTAGACTCGGGGAAGGACAAAATTGATAAGACCAAGTTGGTCACGTACACCAG-----------------------------
                           ***********************************************************************

                         501                                                                                               600
Scaffold12_672601_673490   TAGCGGACTGGACGATGTAATTGCAGCCGCCTCGCCGCAAGCCCCGCATATCAGAAGGTCGTCGGTACGCCACACTTCCTTTTTAGTCTTTTCCAGGCAC
AAE3_01767                 --------------------------CCGCCTCGCCGCAAGCCCCGCATATCAGAAGGTCGTCGG-----------------------------------
                                                     ***************************************

                         601                                                                                               700
Scaffold12_672601_673490   CAACAAGTCACAACCCTTCTCCCACTCCAATAGGCGTCGGCCTCAAAACGGCCGCGGGCTCCATCGTGCCCTACGTCCGGCTCGACATGGACAACACCGG
AAE3_01767                 ----------------------------------CGTCGGCCTCAAAACGGCCGCGGGCTCCATCGTGCCCTACGTCCGGCTCGACATGGACAACACCGG
                                                             ******************************************************************

                         701                                                                                               800
Scaffold12_672601_673490   CAAGGGCATCCATTTCAACGCGACTAAACTCTCCGACAGTTCCGCCAAGCTCGCCGCGGTGCTCAAGACGACGGTGTCCATGACCGAGGCACAGCGAACTC
AAE3_01767                 CAAGGGCATCCATTTCAACGCGACTAAACTCTCCGACAGTTCCGCCAAGCTCGCCGCGGTGCTCAAGACGACGGTGTCCATGACCGAGGCACAGCGAACTC
                           ****************************************************************************************************

                         801                                                                                    889
Scaffold12_672601_673490   AACTCTACATGGAGTATATCAAGGGCATCGAGAATCGGAGTGCGCAGTTTATTTGGGACTGGTGGAGGACGGGCAAGGCTCCGGCGTGA
AAE3_01767                 AACTCTACATGGAGTATATCAAGGGCATCGAGAATCGGAGTGCGCAGTTTATTTGGGACTGGTGGAGGACGGGCAAGGCTCCGGCGTGA
                           *****************************************************************************************
```

Figure S1. Alignment between the genomic region scaffold 12: 672601-673490 (minus strand) and the *ageritin* coding sequence AAE-3_01767. Exons are highlighted in green, introns in red. Putative TATA boxes are underlined.

```
Scaffold12_672601_673490        AGGCGAGAAAAGAAACATAAGACCGCACTCAATGTGAAGGTGATGAATGG
ageritin_gene                   --------------------------------------------------

Scaffold12_672601_673490        TGACTGTCAAAGAAACTTGGGTCATAGGATGAACAAAAGTTTATACCAAC
ageritin_gene                   --------------------------------------------------

Scaffold12_672601_673490        TTTCGAATTTATGGAAGACCTCTGGGGGAGCCTCCGTGAAGTGCAAACGC
ageritin_gene                   --------------------------------------------------

Scaffold12_672601_673490        ACATAAAGAGAAGAGGGAGGTCCTTGAACACGAAAGCCCAGCCCAGAGCT
ageritin_gene                   --------------------------------------------------

Scaffold12_672601_673490        CTCCCAACCACAGGCTCACAAAGCTGTCCTACACAATGTCCGAGTCCTCT
ageritin_gene                   -----------------------------------ATGTCCGAGTCCTCT
                                                                   ***************

Scaffold12_672601_673490        ACCTTCACCACTGCGGTAGTACCTGAAGGTTCTCTCCATCTACTCTTCAT
ageritin_gene                   ACCTTCACCACTGCGGTAGTACCTGAAGGTTCTCTCCATCTACTCTTCAT
                                **************************************************

Scaffold12_672601_673490        CCCTTCCTCTCTCACATGGAACGTCCCTACATGTACAGGCGAAGGAGTTG
ageritin_gene                   CCCTTCCTCTCTCACATGGAACGTCCCTACATGTACAGGCGAAGGAGTTG
                                **************************************************

Scaffold12_672601_673490        CTCCAATGGCAGAGACCGTGCAGTATTACAACTCCTACTCTGACGCATCC
ageritin_gene                   CTCCAATGGCAGAGACCGTGCAGTATTACAACTCCTACTCTGACGCATCC
                                **************************************************

Scaffold12_672601_673490        ATCGCGTCTTGCGCATTTGTAGACTCGGGGAAGGACAAAATTGATAAGAC
ageritin_gene                   ATCGCGTCTTGCGCATTTGTAGACTCGGGGAAGGACAAAATTGATAAGAC
                                **************************************************

Scaffold12_672601_673490        CAAGTTGGTCACGTACACCAGGTAGGCAGGCAGCTTAGAGCGGGAGTGAA
ageritin_gene                   CAAGTTGGTCACGTACACCAGGTAGGCAGGCAGCTTAGAGCGGGAGTGAA
                                **************************************************

Scaffold12_672601_673490        TAGCGGACTGGACGATGTAATTGCAGCCGCCTCGCCGCAAGCCCCGCATA
ageritin_gene                   TAGCGGACTGGACGATGTAATTGCAGCCGCCTCGCCGCAAGCCCCGCATA
                                **************************************************

Scaffold12_672601_673490        TCAGAAGGTCGTCGGTACGCCACACTTCCTTTTTAGTCTTTTCCAGGCAC
ageritin_gene                   TCAGAAGGTCGTCGGTACGCCACACTTCCTTTTTAGTCTTTTCCAGGCAC
                                **************************************************

Scaffold12_672601_673490        CAACAAGTCACAACCCTTCTCCCACTCCAATAGGCGTCGGCCTCAAAACG
ageritin_gene                   CAACAAGTCACAACCCTTCTCCCACTCCAATAGGCGTCGGCCTCAAAACG
                                **************************************************

Scaffold12_672601_673490        GCCGCGGGCTCCATCGTGCCCTACGTCCGGCTCGACATGGACAACACCGG
ageritin_gene                   GCCGCGGGCTCCATCGTGCCCTACGTCCGGCTCGACATGGACAACACCGG
                                **************************************************

Scaffold12_672601_673490        CAAGGGCATCCATTTCAACGCGACTAAACTCTCCGACAGTTCCGCCAAGC
ageritin_gene                   CAAGGGCATCCATTTCAACGCGACTAAACTCTCCGACAGTTCCGCCAAGC
                                **************************************************

Scaffold12_672601_673490        TCGCCGCGGTGCTCAAGACGACGGTGTCCATGACCGAGGCACAGCGAACT
ageritin_gene                   TCGCCGCGGTGCTCAAGACGACGGTGTCCATGACCGAGGCACAGCGAACT
                                **************************************************

Scaffold12_672601_673490        CAACTCTACATGGAGTATATCAAGGGCATCGAGAATCGGAGTGCGCAGTT
ageritin_gene                   CAACTCTACATGGAGTATATCAAGGGCATCGAGAATCGGAGTGCGCAGTT
                                **************************************************

Scaffold12_672601_673490        TATTTGGACTGGTGGAGGACGGGCAAGGCTCCGGCGTGA
ageritin_gene                   TATTTGGACTGGTGGAGGACGGGCAAGGCTCCGGCGTGA
                                ***************************************
```

Figure S2. Alignment between the genomic region scaffold 12: 672601-673490 (minus strand) and the sequence of PCR product obtained by using genomic DNA from *Agrocybe aegerita* (code AA YY) and a couple of primers designed on the basis of *ageritin* coding sequence (Gupta et al., 2020). The 3 exons are highlighted in red.

```
-21                       1                                        40
                          •        •         •          •          •
 MSESSTFTTAVVPEGEGVAPM AETVQYYNSY SDASIASCAF VDSGKDKIDK TKLVTYTSRL

 41                                                                100
 •       •         •          •          •          •          •
 AASPAYQKVV GVGLKTAAGS IVPYVRLDMD NTGKGIHFNA TKLSDSSAKL AAVLKTTVSM

101                                     135
 •       •         •          •    •
 TEAQRTQLYM EYIKGIENRS AQFIWDWWRT GKAPA
```

Figure S3. Amino acid sequence of purified Ageritin (Landi et al., 2019) Histidinyl residue 77 as well as both aspartyl residues 68 and 70 necessary to catalysis are highlighted in red (Ruggiero et al., 2019). The extra N-terminal peptide reported from (Tayyrov et al., 2019) is underlined. This additional peptide plus amino acid sequence of purified Ageritin is named in main text “-$_{+21}$ -Ageritin”. In green, asparaginyl residues potential *N*-glycosylation sites.

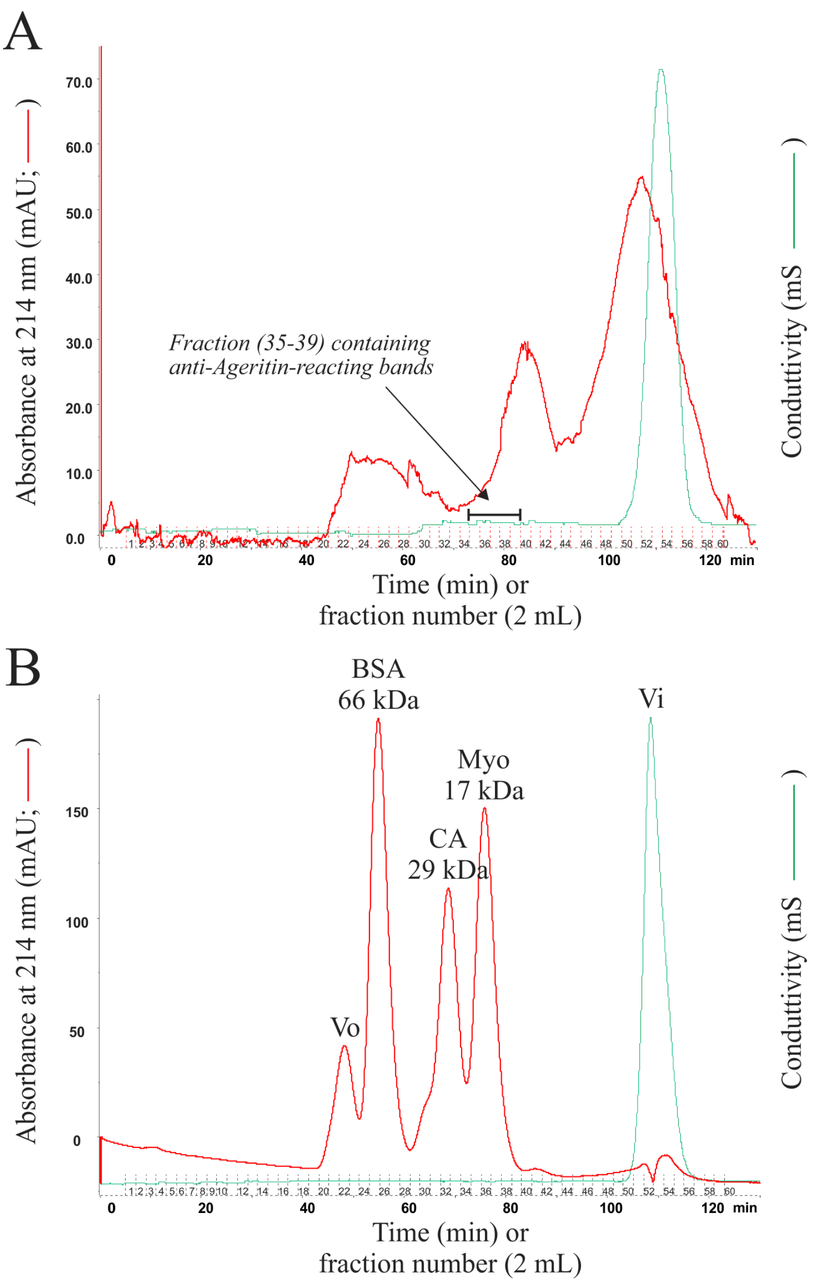

Figure S4. (A) Raw elution profile from gel-filtration chromatography on a HiLoad 16/60 Superdex 75 column obtained from mycelium basic proteins (see paragraph 2.2 main text). (B) Elution profile of standard proteins. V_0 and V_i, void and included volumes, respectively. BSA, Bovine Serum A (66 kDa). CA, carbonic anhydrase (29 kDa), Myo, horse myoglobin (17 kDa).
